# Effect of the Viral Hemorrhagic Septicemia Virus Nonvirion Protein on Translation via PERK-eIF2α Pathway

**DOI:** 10.3390/v12050499

**Published:** 2020-04-30

**Authors:** Shelby Powell Kesterson, Jeffery Ringiesn, Vikram N. Vakharia, Brian S. Shepherd, Douglas W. Leaman, Krishnamurthy Malathi

**Affiliations:** 1 Department of Biological Sciences, University of Toledo, 2801 West Bancroft Street, Toledo, OH 43606, USA; shelby.powell@rockets.utoledo.edu; 2 Department of Biological Sciences, Wright State University, 3640 Colonel Glenn Hwy, Dayton, OH 45435, USA; jeff.ringiesn@wright.edu; 3 Institute of Marine and Environmental Technology, University of Maryland Baltimore County, Baltimore, MD 21202, USA; vakharia@umbc.edu; 4 USDA/ARS, School of Freshwater Sciences, University of Wisconsin—Milwaukee, Milwaukee, Wisconsin, WI 53204, USA; brian.shepherd@usda.gov

**Keywords:** VHSV, nonvirion, interferon, PERK, eIF2α phosphorylation, translation

## Abstract

Viral hemorrhagic septicemia virus (VHSV) is one of the most deadly infectious fish pathogens, posing a serious threat to the aquaculture industry and freshwater ecosystems worldwide. Previous work showed that VHSV sub-genotype IVb suppresses host innate immune responses, but the exact mechanism by which VHSV IVb inhibits antiviral response remains incompletely characterized. As with other novirhabdoviruses, VHSV IVb contains a unique and highly variable nonvirion (NV) gene, which is implicated in viral replication, virus-induced apoptosis and regulating interferon (IFN) production. However, the molecular mechanisms underlying the role of IVb NV gene in regulating viral or cellular processes is poorly understood. Compared to the wild-type recombinant (rWT) VHSV, mutant VHSV lacking a functional IVb NV reduced IFN expression and compromised innate immune response of the host cells by inhibiting translation. VHSV IVb infection increased phosphorylated eukaryotic initiation factor 2α (p-eIF2α), resulting in host translation shutoff. However, VHSV IVb protein synthesis proceeds despite increasing phosphorylation of eIF2α. During VHSV IVb infection, eIF2α phosphorylation was mediated via PKR-like endoplasmic reticulum kinase (PERK) and was required for efficient viral protein synthesis, but shutoff of host translation and IFN signaling was independent of p-eIF2α. Similarly, IVb NV null VHSV infection induced less p-eIF2α, but exhibited decreased viral protein synthesis despite increased levels of viral mRNA. These findings show a role for IVb NV in VHSV pathogenesis by utilizing the PERK-eIF2α pathway for viral-mediated host shutoff and interferon signaling to regulate host cell response.

## 1. Introduction

Viral hemorrhagic septicemia virus (VHSV), also known as Piscine novirhabdovirus, is one of the world’s most deadly infectious fish pathogens, with a mortality rate as high as 100% in juvenile fish. VHSV infects more than 90 marine and freshwater species worldwide, posing a serious threat to the aquaculture industry. VHSV belongs to the *Rhabdoviridae* family and the presence of a small, unique, and highly variable nonvirion (NV) protein further categorizes VHSV to the genus *Novirhabdovirus* [1]. VHSV is a bullet-shaped, enveloped virion containing a non-segmented, negative sense, single stranded RNA genome of approximately 11 kb that codes for five structural proteins; nucleoprotein (N), phosphoprotein (P), matrix protein (M), glycoprotein (G), a RNA-dependent RNA polymerase (L) and a nonstructural protein (NV). The VHSV gene order is 3′-leader-N-P-M-G-NV-L-trailer-5′ [2,3,4]. VHSV has been classified into four individual genotypes (I-IV) based on geographic location and genomic sequence similarities of G and N genes [5,6]. Genotype I is further divided into five sub lineages (Ia to Ie) and genotype IV is further divided into three sub lineages (IVa to IVc). VHSV IVb was first identified in Lake St. Clair in the Great Lakes region in 2003 and has since been identified in all five of the Great Lakes. In 2005, VHSV IVb strain was detected in the Great Lakes as a major cause of mortality in the naïve fish population, posing a threat to farmed fish. Further, VHSV IVb provides a new model to study invasive virus species and the mechanism of virulence of viruses that pose threat to the aquaculture industry [7,8,9,10].

Similar to other rhabdoviruses, VHSV manipulates host innate immune responses to ensure efficient viral replication. Infected host cells recognize viral RNA as a foreign, conserved pathogen-associated molecular pattern (PAMP) via germ line-encoded pattern recognition receptors (PRRs) such as the retinoic acid-inducible gene 1 (RIG-I)-like helicases (RLHs), which include RIG-I, melanoma differentiation-associated factor 5 (MDA5), and laboratory of genetics and physiology 2 (LGP2) [11,12,13]. Viral RNA detection leads to the activation of the integrated stress response (ISR), which in turn activates a variety of antiviral innate immune pathways, including the type I interferon (IFN) pathway. Upon activation, both RIG-I and MDA5 recruit and activate the mitochondrial antiviral signaling (MAVS) protein, which in turn leads to the activation of downstream signaling molecules and induction of type I IFNs [14,15,16,17]. Type I IFNs produced by infected cells bind to the cognate type I IFN receptor (IFNAR) complex on neighboring cells, resulting in the activation of signal transducer and activator of transcription (STAT)-dependent signaling cascades, thus leading to the upregulation of interferon-stimulated genes (ISG) [18]. These ISGs encode for a variety of proteins that can impact cellular functions, including transcriptional and translational regulation that can establish an antiviral state in the cell [19].

Viruses depend entirely on the host-cell protein synthesis machinery for the production of viral proteins and have developed a variety of mechanisms to effectively translate viral mRNAs and to inhibit cellular host mRNA translation to evade the innate immune response [20]. Host cells in turn activate phosphorylation of eukaryotic initiation factor 2α (eIF2α), which globally blocks translation of both cellular and viral RNAs. The activation of four different eIF2α kinases; double stranded RNA (dsRNA)- dependent protein kinase (PKR), RNA-regulated protein kinase (PKR)-like endoplasmic reticulum kinase (PERK), general control non-depressible gene 2 kinase (GCN2) and heme-regulated kinase (HRI) can lead to the phosphorylation of eIF2α in response to different stressors [21]. PKR and PERK are the two key kinases activated in response to viral infection and can prevent viral replication by inhibiting translation. PKR autophosphorylates in response to binding to both cellular and viral dsRNA via two dsRNA binding domains (dsRBD), which leads to the phosphorylation of eIF2α [22]. The endoplasmic reticulum (ER) is an important organelle for viral replication and maturation. In response to the accumulation of unfolded or misfolded proteins, PERK phosphorylates eIF2α to inhibit general protein synthesis to try to reduce the protein load in the ER and ultimately reduce ER stress [23].

Previous studies have implicated the VHSV M protein as a primary suppressor of host antiviral responses, specifically at the transcriptional level [24]. IVb NV was also implicated in the possible augmentation of IFN signaling during VHSV infection, while other studies have demonstrated a possible role of Ia and IVa NV in the suppression of innate and adaptive immune related genes [25,26,27,28,29]. IVa NV suppresses TNFα-mediated NF-κB activation and Ia NV was shown to recruit protein phosphatase, Mg^2+^/Mn^2+^-dependent 1Bb (PPM1Bb) to counteract RIG-I- and Traf family member-associated NF-κB (TANK)-binding kinase (TBK1)-dependent IFN and ISG activation [27]. Deletion of IVb NV led to earlier onset of viral-induced apoptosis and suggested that IVb NV was necessary for efficient virus replication in cell culture, but was not a determinant in host specific virulence [30,31,32,33]. Mass spectroscopy studies showed that Ia NV protein interacted with other viral proteins along with at least 35 host proteins, including a variety of RNA binding proteins involved in RNA processing, RNA transcription, RNA localization and regulation of gene expression as well as proteins involved with replication and transport of the viral genome. It also interacted with a variety of host proteins involved in host defense against virus infection, including regulation of stress response, IFNβ production and apoptosis, suggesting that NV may play a broader role in VHSV infection and cellular host and stress responses than previously believed [25]. However, the exact mechanism by which NV impacts VHSV infection and the host cell remains poorly characterized.

In this study, we assessed the role of IVb NV in regulating host antiviral responses and VHSV replication. IVb NV deletion resulted in significantly decreased IFN induction and response and host translation shutoff. Furthermore, infection of VHSV IVb NV mutant viruses caused decreased viral protein expression and viral yield, despite an increase in viral mRNA. VHSV IVb infection activated the PERK pathway, which mediated phosphorylation of eIF2α. Interestingly, VHSV lacking IVb NV protein was less able to promote eIF2α phosphorylation. Blocking PERK-mediated p-eIF2α with a pharmacological inhibitor resulted in decreased viral protein expression as well as a decrease in viral yield, but not a rescue of host translation or IFN signaling. These data suggest a critical role of VHSV IVb NV in VHSV-induced phosphorylation of eIF2α by PERK and regulation of viral-mediated host shutoff.

## 2. Materials and Methods

### 2.1. Cell Lines and Culture Conditions

Epithelioma papulosum cyprinid (EPC) and Bluegill Fry (BF-2) cells were purchased from the American Type Culture Collection (ATCC, Rockville, MD, USA; CRL-2872/ CCL-91, respectively). The cells were grown in Hyclone defined L-15 Leibovitz media supplemented with 10% fetal bovine serum (FBS) (Sigma-Aldrich, St. Louis, MO, USA) and 1% penicillin-streptomycin (Invitrogen, Thermo Fisher Scientific, Waltham, MA, USA) (complete L-15) at 20°C without CO_2_.

### 2.2. VHSV Infection

EPC cells were infected with VHSV in serum-free L-15 at a multiplicity of infection (MOI) of 1, unless otherwise stated. After 1.5 h, the virus-containing medium was removed, and cells were washed with phosphate-buffered saline (PBS) and replenished with complete L-15 media.

### 2.3. VHSV Amplification and Purification

The recombinant Great Lakes VHSV strain (MIO3GL), rVHSV-∆NV, and the rVHSV-∆ATG were described previously [30]. Briefly, rVHSV-∆ATG was created by mutating the two ATG codons of the IVb NV open reading frame to TAG, and rVHSV-∆NV was created by deleting the entire open reading frame of the IVb NV gene. Recombinant virus stocks were prepared by infecting confluent EPC cells with a 1:1000 (*v*/*v*) dilution of unpurified virus stock from BF-2 infected cells in serum-free L-15. After 1.5 h, the virus-containing medium was replaced with complete L-15. Following the onset of cytopathicity (72–96 h), the virus-containing media and attached cells were subjected to a freeze–thaw cycle. Cellular debris was removed by centrifugation (6000 rpm, 20 min, 4 °C) and then clarified using a 0.22 µm syringe tip filter. Resulting supernatant was then subjected to ultracentrifugation through 25% Sucrose weight by volume (*w*/*v*) in TEN buffer (10 mM Tris HCl pH 7.5, 150 mM NaCl, 1 mM EDTA) at 25,000 rpm for 2 h at 4 °C. The virus-containing pellet was resuspended in phosphate-buffered saline (PBS) and stored at −80 °C until use.

### 2.4. Chemicals, Plasmids and Antibodies

Cycloheximide and the PKR inhibitor C16 were purchased from Sigma-Aldrich (St. Louis, MO, USA); the PKR inhibitor 2-Aminopurine (2-AP) from InvivoGen (San Diego, CA, USA); the PERK inhibitor GSK2656157 from Santa Cruz Biotechnology (Dallas, TX, USA); puromycin from Fisher Scientific (Hampton, NH, USA). Puromycin was added to cells at indicated time points for 20 min prior to lysate collection. Inhibitors were added at indicated concentrations 1 h prior to infection and added post viral adsorption.

The EPC MAVS expression plasmid was obtained from Michel Bremont (French National Institute for Agricultural Research, Jouy-en-Josas, France), the IFNβ-luciferase reporter was obtained from John Hiscott (Istituto Pasteur Fondazione Cenci Bolognetti, Rome, Italy), the p56-luciferase reporter (human) from Ganes Sen (Cleveland Clinic, Cleveland, OH, USA), and the MX1-luciferase reporter was described previously [34]. The EPC IFN (fIFN) was obtained from cultured supernatants of EPC overexpressing MAVS and units of IFN were determined by IFN bioassay and applied to cells. The construction of VHSV NV plasmid has been described previously [24]. VHSV-ATG target coding sequences were PCR cloned from viral stocks using specific primers (Table 1). PCR fragments were cloned into pcDNA3.1(-) Myc/His A (Invitrogen, Thermo Fisher Scientific, Waltham, MA, USA).

Mouse anti-myc (#2276S) and rabbit anti-p-eIF2α (S51) (#3398) were purchased from Cell Signaling Technology (Danvers, MA, USA); Mouse anti-puromycin was purchased from Sigma Aldrich (St. Louis, MO, USA) and rabbit anti-eIF2S1 and mouse anti-β-actin was purchased from Proteintech (Rosemont, IL, USA). Rabbit anti-VHSV was obtained from Vikram Vakharia (University of Maryland Baltimore County, Baltimore, MD, USA). All primary antibodies were used at 1:1000 in 5% (*w*/*v*) BSA/TBST.

### 2.5. Virus Yield and IFN Bioassays

To assess viral replication and IFN secretion, the media were harvested at indicated time points following infection of EPC cells with the indicated viruses. Virus yield was determined by applying 1:10 serial dilutions in SF L-15 of the harvested media to EPC cells. Following viral adsorption, a carboxymethyl-cellulose (CMC) overlay consisting of 0.5% CMC and 2% FBS was added. Following onset of cytopathicity (72–96 h), infected cells were fixed with 10% formalin and stained with crystal violet. Viral plaques were counted and a final viral concentration (PFU/mL) was calculated for each time point. IFN bioassays were used to assess IFN production during viral infection. Briefly, 1:3 dilutions of the UV-irradiated medium from the indicated samples were applied to EPC cells for 24 h. Following treatment with the irradiated medium, cells were infected with rWT VHSV (MOI 0.1) for 72 h and then fixed with methanol and stained with crystal violet. One unit of IFN activity was determined by the dilution necessary to provide 50% protection from virus CPE.

### 2.6. Transfection

EPC cell transfections were performed using FuGENE (Promega, Madison, WI, USA) according to the manufacturer’s instructions. Plasmids were mixed with FuGENE in serum-free L-15 for 10 min at room temperature and then added to cells in the serum-free medium. The complete medium was added to cells after 3 h of incubation.

### 2.7. Luciferase Assays

EPC cells (4 × 10^5^) were transfected with the indicated plasmids for 24 or 48 h, washed with PBS twice and then lysed for 15 min at 4 °C with 100 µL of 1× passive lysis buffer (Promega, Madison, WI, USA). Fifty µL of the clarified lysate was combined with 50 µl of luciferase buffer (250 mM Glycylglycine, 200 mM DTT, 100 mM ATP, 200 mM luciferin) and relative luminescence values determined using Molecular Device SpectroMax ID5 (Molecular Devices, LLC, San Jose, CA, USA). Luciferase values were normalized to total protein content of each sample determined by Bradford assay.

### 2.8. Immunoblotting

Cells were lysed with NP-40 lysis buffer (0.5% NP-40, 90 mM KCl, 5 mM magnesium acetate, 20 mM Tris, pH 7.5, 5 mM β mercaptoethanol, 0.1 M phenylmethylsulfonyl fluoride (PMSF), 0.2 mM sodium orthovanadate, 50 mM NaF, 10 mM glycerophosphate, protease inhibitor (Roche Diagnostics, Indianapolis, IN, USA)). Samples were separated by SDS-PAGE and electrophoretically transferred to PVDF membrane (Immobilon; Millipore, Burlington, MA, USA). Membranes were blocked with 10% (*w*/*v*) nonfat dry milk/TBST for 2 h at room temperature. Primary antibodies were diluted in 5% (*w*/*v*) BSA/TBST (P-753, Boston Bioproducts, Ashland, MA, USA) and incubated overnight at 4 °C. The membrane was then incubated with a secondary antibody in TBST for 2 h at room temperature (Cell Signaling Technology, Danvers, MA, USA; Anti-mouse IgG, HRP-linked antibody #7076 and anti-rabbit IgG, HRP linked antibody #7074). Immunoreactive bands were visualized with ECL (Boston Bioproducts, Ashland, MA, USA) using a ChemiDoc-It^2^ 510 imager (UVP, Fisher Scientific; Hampton, NH, USA).

### 2.9. Click-iT OPP Alexa Fluor 488 Imaging and Immunofluorescence Microscopy

Protein synthesis was detected in cells using fluorescence microscopy and a Click-iT OPP protein synthesis assay kit (Invitrogen, Thermo Fisher Scientific, Waltham, MA, USA), following the manufacturer’s instructions. EPC cells were seeded onto Poly-L-Lysine (0.01% *w*/*v*) coated coverslips (Sigma-Aldrich, St. Louis, MO, USA) and left uninfected or infected for indicated time points. Cells were treated in parallel with cycloheximide (CHX), a well-established translation inhibitor, as a positive control for translational inhibition. At designated time points, Click-iT OPP reagent was added to cells at a final concentration of 20 µM for 30 min. Cells were fixed with 4% paraformaldehyde (BM-155; Boston Bioproducts, Ashland, MA, USA) for 15 min at room temperature and then permeabilized with 0.5% Triton X-100 in PBS for 15 min at room temperature. Click-iT Plus reaction cocktail was made fresh and added to cells for 30 min at room temperature. Cells were then blocked with 3% BSA, 0.02% Tween in PBS for 1 h at room temperature and then incubated for 2 h with the indicated antibodies diluted in 3% BSA. Cells were then incubated with a secondary antibody (Alexa 488- or Alexa 647-conjugated anti-immunoglobulin antibody, Molecular Probes; Eugene, OR, USA) for 1 h at room temperature. Cell nuclei were stained with DAPI (17985–50; EMS, Hatfield, PA, USA). Cells were imaged on an Olympus IX81 inverted fluorescence microscope and the analysis and processing of images were performed using Stream View and ImageJ software [35]. Incorporation of OPP as an indicator of protein translation was normalized to levels of VHSV staining and compared to uninfected cells in at least 30 cells from various fields. Data are representative of two independent experiments.

### 2.10. Real-Time Quantitative PCR

Gene expression was determined by quantitative reverse transcription polymerase chain reaction (qRT-PCR) using total RNA reverse transcribed to cDNA that was used as template for qPCR reaction using gene-specific primers. RNA was isolated using TRIzol (Invitrogen, Thermo Fisher Scientific, Waltham, MA, USA) according to manufacturer’s protocol. Total RNA was reverse transcribed using Moloney murine leukemia virus (M-MLV) reverse transcriptase (Promega, Madison, WI, USA). Reverse transcription reactions were carried out by incubating RNA with 100 ng of random hexamer primer and nuclease-free water at 70 °C for 10 min, then, briefly cooled on ice before the addition of 5× MMLV RT buffer, MMLV RT, RNAse inhibitor and dNTPs (Fisher Scientific, Hampton, NH, USA). Samples were incubated at 42 °C for 1 h followed by a 10 min incubation at 92 °C. Viral RNA samples were spiked with 1 ng of in vitro-transcribed GFP RNA to serve as an internal control for normalization. cDNA samples were diluted 1:10 with water and then quantified with quantitative reverse transcription PCR (qRT-PCR) using 5 µL of 2× SYBR qPCR Master Mix (1725120, Bio-Rad, Hercules, CA, USA), 2 µL of diluted cDNA, 50 ng of each primer and water to a total volume of 10 µL. Reactions and data collection were performed with a Bio-Rad C1000 real-time thermocycler for 3 min of initial denaturation at 95 °C, followed by 40 cycles of 15 s at 95 °C for denaturation and 30 s at 60 °C for elongation. Cycle threshold (C_t_) values were obtained by an automated single point threshold within the log-linear range and normalized to the spiked internal control GFP. Expression levels were calculated using the 2^−∆∆*CT*^ method. VHSV targeted primers were designed using GenBank accession GQ375941. The primer sequences used are listed in Table 2.

### 2.11. Statistics

Data management, analysis, and graphing were done using Prism7 software (GraphPad). Data were analyzed by two-tailed, unpaired Student *t* tests and are presented as the mean ± standard error of mean (SEM). All data are representative of at least three independent experiments.

## 3. Results

### 3.1. VHSV IVb NV Protein Augments IFN Signaling

**Figure 1:**
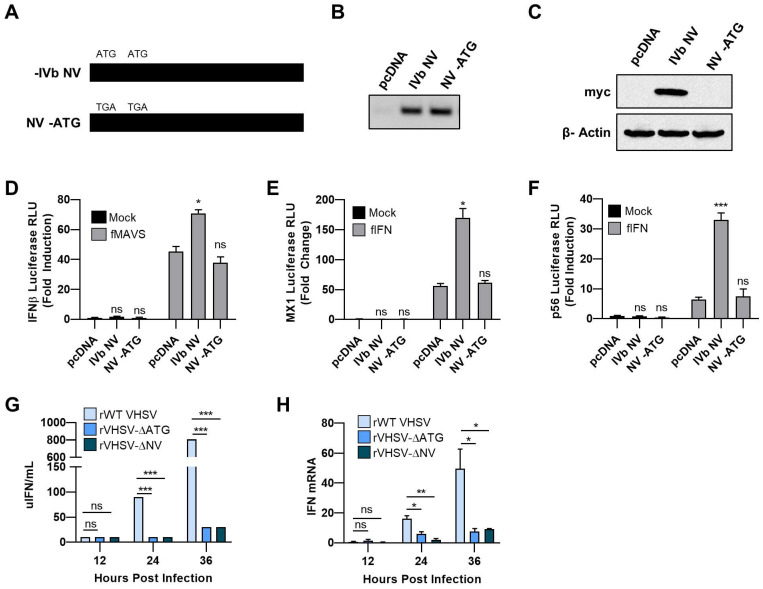
VHSV IVb NV protein augments IFN signaling. (**A**) Schematic of the VHSV IVb NV mutants (B,C) EPC cells (1.5 × 10^6^) were transfected with 3 µg of empty plasmid, VHSV NV and NV-ATG with C-terminal Myc epitope tag for 48 h. (**B**) RT-PCR analysis of NV mRNA expression. (**C**) EPC cell lysates were separated by SDS-PAGE and immunoblotted for Myc-NV protein expression with Myc antibodies. (**D**) EPC cells were cotransfected with 0.4 µg of human IFN-β (hIFNβ)/*luc* construct (0.4 µg), MAVS (0.2 µg), and plasmids encoding the VHSV NV and NV-ATG (0.1 µg), followed by luciferase assay 24 h later. Luciferase values were normalized to hIFNβ/*luc* without MAVS and compared to pcDNA. (**E**) EPC cells were cotransfected with 0.4 µg of MX1/*luc* and plasmids encoding the VHSV NV and NV-ATG (0.1 µ) for 24 h, followed by EPC IFN treatment. Luciferase values were quantified after and normalized to MX1/*luc* without IFN treatment. (**F**) EPC cells were cotransfected with 0.4 µg of human p56/*luc* and plasmids encoding the VHSV NV and NV-ATG (0.1 µg) for 24 h, followed by EPC IFN treatment. Luciferase values were quantified 24 h later. Luciferase values were normalized to p56/*luc* without IFN treatment and compared to pcDNA. (**G**) Media collected at the indicated times post infection were used to measure units of IFN (uIFN/mL) produced during the course of viral infection. (**H**) EPC cells were infected with the indicated virus for 12, 24, or 36 h, followed by RNA isolation and IFNβ mRNA levels determined by RT-PCR. Data were normalized to spiked internal control and presented as fold change in expression compared to non-infected. Error bars reflect SEM *, *p* < 0.05; **, *p* < 0.01; ***, *p* < 0.001.

Previous studies demonstrated a role for VHSV IVb M protein in inhibition of IFN signaling. In contrast, VHSV IVb NV protein expression positively regulated IFN signaling [24]. To better examine the role of VHSV IVb NV on IFN signaling, we compared the effect of WT IVb NV to NV-ATG, in which the two ATG start codons of the IVb NV open reading frame were mutated to TGA codons (NV-ATG) on IFN-β promoter induction (Figure 1A–C). While WT IVb NV expression resulted in 25-fold increase in IFN-β promoter activity, NV-ATG did not alter IFN-β promoter activity, suggesting an impact of the IVb NV protein alone on IFN production (Figure 1D). We further determined the impact of IVb NV on interferon signaling and interferon-stimulated gene (ISG) expression by assessing MX1 promoter activity in luciferase assays. We observed a 3-fold increase in promoter activity with WT NV cotransfection as compared to the control plasmid or NV-ATG cotransfection (Figure 1E). A similar increase in p56 promoter activity was observed upon WT, but not NV-ATG cotransfection, suggesting a broad augmentation of innate immune signaling by the IVb NV protein (Figure 1F).

To further study the impact of IVb NV on innate immune signaling during viral infection, we utilized wild type recombinant VHSV IVb (rWT VHSV), a mutant recombinant virus in which the two ATG codons of the IVb NV open reading frame were mutated to TAG (rVHSV-∆ATG), or a mutant virus in which the entire open reading frame of the IVb NV gene was deleted (rVHSV-ΔNV). To investigate the impact of IVb NV deletion on IFN production, we measured IFN production by infected cells, utilizing an IFN bioassay (Figure 1G). rWT VHSV IVb produced significantly more IFN during the course of infection compared to infection with the two IVb NV deficient viruses. RNA harvested during a time course viral infection was assessed for IFN mRNA levels (Figure 1H). rWT VHSV IVb infection resulted in a 60-fold increase in IFN mRNA compared to both VHSV-∆ATG and VHSV-∆NV. Together, these results indicate that VHSV IVb NV augments IFN signaling and VHSV lacking IVb NV reduced IFN upregulation.

### 3.2. IVb NV Deficient VHSV Infection Inhibits IFN Signaling

**Figure 2:**
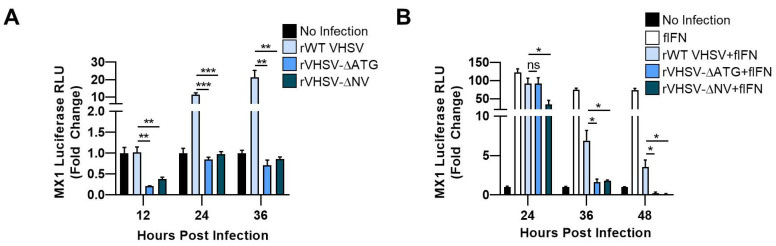
NV deficient VHSV inhibits IFN signaling. (**A**) EPC cells were transfected with MX1/*luc* for 24 h, followed by infection with the indicated virus for 12, 24, or 36 h. Luciferase assay values were quantified and normalized to non-infected. (**B**) EPC cells were transfected with MX1/*luc* for 24 h, followed by infection with the indicated virus for 24, 36, or 48 h. Infected cells were treated with EPC IFN and luciferase assay values were quantified 12 h later and normalized to non-infected. Error bars reflect SEM *, *p* < 0.05; **, *p*< 0.01; ***, *p*< 0.001.

To determine if the decrease in IFN transcription observed in cells infected with IVb NV null virus compared to WT virus was due to a decrease in the antiviral response to infection or an increase in the shutdown of the host antiviral response by IVb NV null VHSV, we assessed the impact of VHSV IVb infection on MX1 promoter/luciferase activity with and without exogenous IFN treatment. As expected, rWT VHSV IVb infection alone induced the MX1 promoter 20-fold more than VHSV lacking IVb NV protein (VHSV-ΔNV) (Figure 2A). When cells were instead treated with exogenous IFN after virus infection, MX1 promoter activity was reduced by rWT VHSV IVb infection compared to IFN treatment alone at 36 or 48 h post infection (Figure 2B). However, both of the IVb NV deficient viruses decreased MX1 promoter activity more robustly than rWT VHSV IVb (Figure 2B). These data suggest that IVb NV-null VHSV is more capable of inhibiting the host antiviral responses as compared to rWT VHSV IVb, implicating IVb NV in suppressing the anti-host effects of the VHSV genes during the course of a normal infection.

### 3.3. IVb NV Is Required for Efficient Viral Protein Synthesis

**Figure 3:**
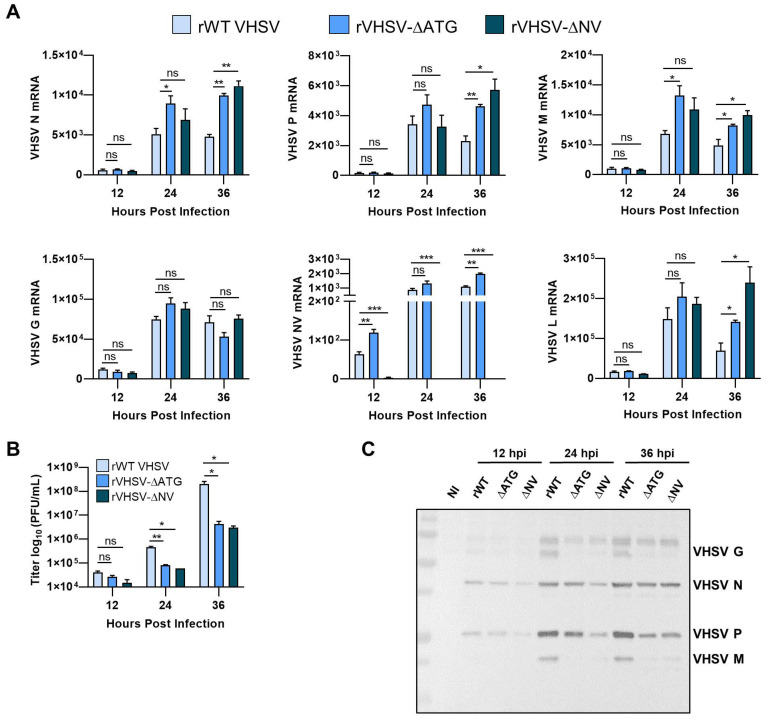
Effect of VHSV NV on viral protein synthesis. (**A**) RT-qPCR analysis of mRNA levels of VHSV N, VHSV P, VHSV M, VHSV G, VHSV NV, and VHSV L. Graphed as fold change normalized to no infection (NI). (**B**) Media collected at the indicated times post infection were used to determine viral titers. Error bars reflect SEM *, *p* < 0.05; **, *p*< 0.01; ***, *p*< 0.001. (**C**) EPC cells (1.5 × 10^6^) were infected with the indicated virus for various times as at a MOI of 1. Cell lysates were separated by SDS-PAGE and immunoblotted for viral protein expression with VHSV antibodies.

Removal of IVb NV from recombinant VHSV leads to decreased viral protein expression and a decrease in viral yield [18]. To assess the impact of IVb NV on viral-dependent transcription, RNA harvested at indicated times post infection was assessed for levels of viral mRNAs (Figure 3A). We observed a small but significant increase in levels of VHSV N, P, M, NV, and L mRNA during both VHSV-∆ATG and VHSV-∆NV viral infection compared to rWT VHSV IVb. There was no significant difference in levels of viral mRNA between the two NV mutant viruses. Unlike other viral mRNAs, we did not observe significant difference in levels of VHSV G mRNA. However, despite the slight increase in viral mRNA levels, we observed a log decrease in viral yield as well as decreased expression of viral proteins with IVb NV mutant VHSV infection (Figure 3B,C). The decrease in viral protein expression, despite the increase in viral mRNA levels, suggest that IVb NV may play a critical role in the synthesis of viral proteins.

### 3.4. IVb NV Deficient VHSV Inhibits Translation

**Figure 4:**
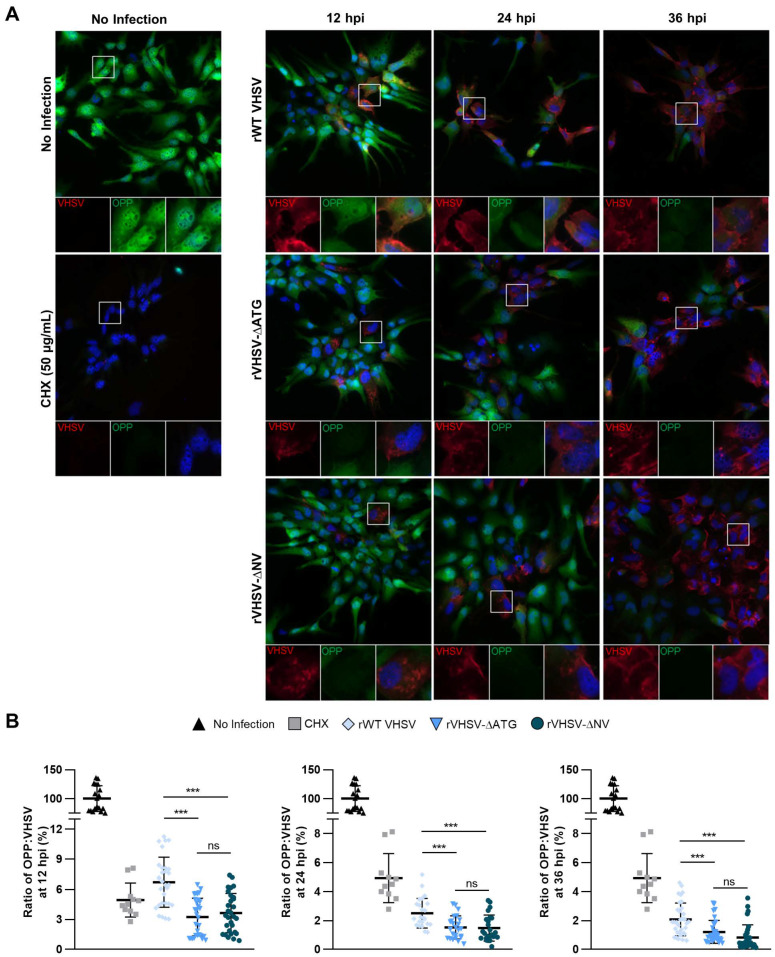
NV deficient VHSV inhibits translation. (**A**) EPC cells were infected with rWT VHSV, VHSV-∆ATG, and VHSV-∆NV for 12, 24, or 36 h at a MOI 1. At designated time points, levels of newly synthesized proteins in infected cells were visualized by Click-iT OPP Alexa Fluor 488 imaging and immunofluorescence analysis. Image magnification 60× (**B**) Quantification of (A). OPP fluorescence signal normalized to VHSV fluorescence and plotted as a percent ratio normalized to no infection. Error bars reflect SD *, *p* < 0.05; **, *p* < 0.01; ***, *p* < 0.001.

Viruses must compete with the host to ensure translation of their own mRNAs into viral proteins preferentially over host mRNAs translation into protein. The impact of VHSV IVb on protein translation was determined by measuring incorporation of puromycin and normalized to VHSV levels for indicated times and compared with uninfected cells using immunofluorescence microscopy (Figure 4A,B). At 12 hpi, we observed a 93% decrease in OPP incorporation with rWT VHSV IVb infection compared to no infection. This decrease continued into the later time points (Figure 4A,B). These results suggest that VHSV IVb infection results in a significant decrease in translation, but still allows viral protein synthesis to occur (see Figure 3C). IVb NV deficient VHSV infection also resulted in a significant decrease in OPP incorporation (97% and 99%) compared to no infection, suggesting the removal of IVb NV did not inhibit the ability of VHSV to inhibit host translation (Figure 4A,B). Indeed, removal of IVb NV resulted in significantly lower new protein synthesis as compared to rWT VHSV IVb. These results suggest that removal of IVb NV may inhibit the ability of VHSV to overcome the inhibition of host translation and synthesize its own viral proteins.

### 3.5. VHSV IVb Infection Inhibits Host Translation and Phosphorylates eIF2α

**Figure 5:**
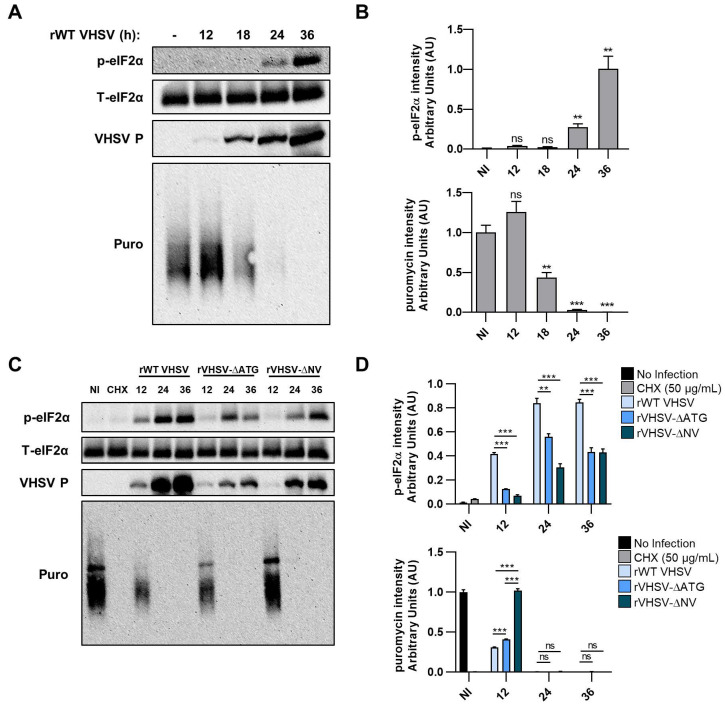
VHSV infection inhibits host translation and phosphorylates eIF2α. (**A**) EPC cells (1.5 × 10^6^) were infected with rWT VHSV at a MOI of 1. At indicated time points, cells were pulsed with puromycin (10 µg/mL) for 20 min and cell lysates were separated by SDS-PAGE and immunoblotted for p-eIF2α, T-eIF2α, puromycin and viral proteins. (**B**) Quantification of (A). p-eIF2α and puromycin signal normalized to T-eIF2α signal and graphed as a ratio. (**C**) EPC cells (1.5 × 10^6^) were infected with rWT VHSV, VHSV-∆ATG or VHSV-∆NV for indicated time points at a MOI of 1. Cell lysates were separated by SDS-PAGE and immunoblotted for p-eIF2α, T-eIF2α and viral proteins. (**D**) Quantification of (**C**). p-eIF2α signal normalized to T-eIF2α and graphed as a ratio. Error bars reflect SEM *, *p* < 0.05; **, *p* < 0.01; ***, *p* < 0.001. Samples compared to non-infected.

Stress response pathways, including virus infection, result in phosphorylation of eIF2α causing shutoff of host protein translation. To determine the role of VHSV IVb on translation, we examined the phosphorylation of eIF2α during infection. EPC cells were infected for indicated times and immunoblot analysis showed increased phosphorylation of eIF2α, whereas, no change in levels of total eIF2α was observed (Figure 5A,B). Phosphorylation of eIF2α increased significantly at 24 hpi and continued to 36 hpi. Viral protein expression was observed as early as 12 hpi and gradually increased at 36 hpi (Figure 5A,B). These results suggest that VHSV IVb infection promotes phosphorylation of eIF2α as viral proteins are expressed. To examine the impact of increased phosphorylation of eIF2α on translation of cellular proteins, EPC cells were infected with VHSV IVb for indicated times and pulsed with puromycin for 20 min to monitor newly translated proteins prior to making cell lysates (Figure 5A). Puromycin incorporates into newly translated polypeptides and terminates translation, allowing analysis of newly synthesized protein with anti-puromycin antibodies on immunoblots. We treated cells with CHX as a positive control. Immunoblot analysis using anti-puromycin antibodies showed increase in incorporation, indicating new protein synthesis, at 12 hpi (Figure 5A,B). At later infection times, the levels of puromycin incorporated into proteins decreased significantly, despite the increase in VHSV P synthesis (Figure 5A,B). Taken together, these results indicate that VHSV-induced phosphorylation of eIF2α corresponded closely with a decrease in host cell protein synthesis, while viral protein synthesis continued to increase during the shutoff of host cell translation.

To better understand the impact of IVb NV protein on host translation, we compared phosphorylation of eIF2α in EPC cells infected with rWT VHSV IVb or VHSV IVb NV mutant viruses. Levels of p-eIF2α in IVb NV mutant virus infection were significantly lower at 12 hpi as compared to rWT VHSV (Figure 5C,D). Interestingly, the puromycin incorporation in IVb NV deleted VHSV infected EPC cells was comparable to uninfected cells, although the ΔATG mutant was similar to WT virus (Figure 5C,D). Interestingly, the puromycin incorporation in IVb NV-deleted VHSV was comparable to uninfected cells. However, at later times of infection, translational shutoff correlated with p-eIF2α in all three virus infections. Likewise, consistent with our previous observations (Figure 3C), we see a decrease in the accumulation of viral proteins in VHSV IVb NV mutant viruses compared to rWT VHSV IVb.

### 3.6. Inhibiting PERK Abolishes VHSV IVb-Induced Phosphorylation of eIF2α But Does Not Rescue Host Translation

**Figure 6:**
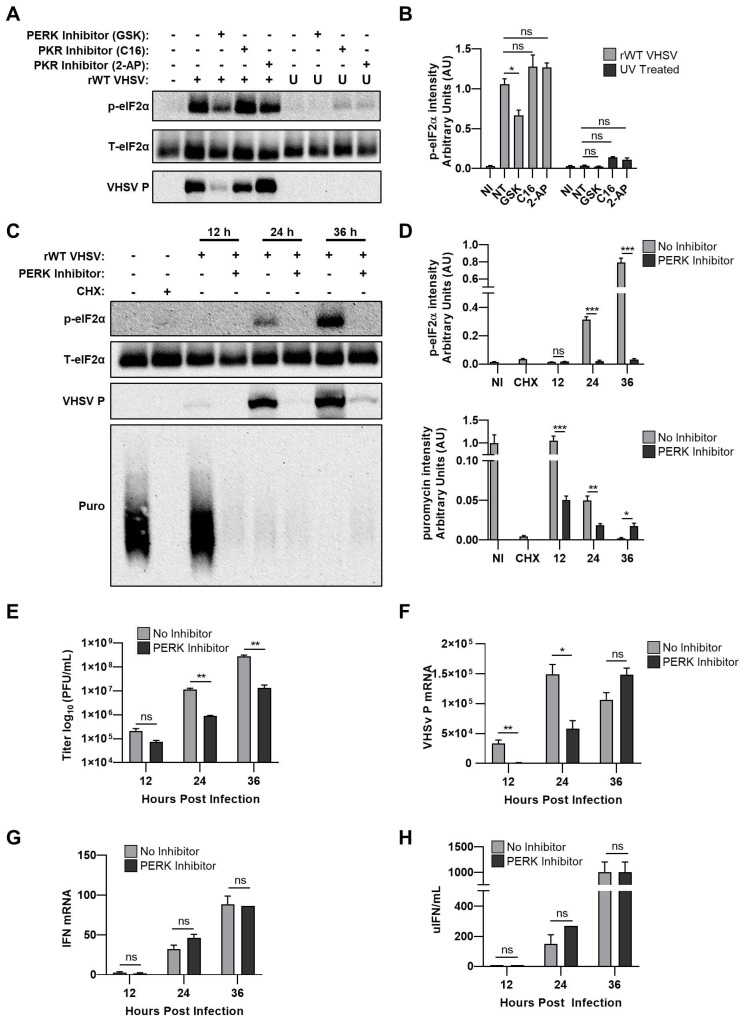
Inhibiting PERK pathway abolishes VHSV-induced phosphorylation of eIF2α but does not rescue host translation. (**A**) EPC cells (1.5 × 10^6^) were treated with GSK2656157 (5 µM), C16 (1 µM), or 2-AP (10 mM) at 1 h prior to infection with rWT VHSV or UV-irradiated rWT VHSV for 24 h before cell lysate samples were obtained. Cell lysates were separated by SDS-PAGE and immunoblotted with antibodies against VHSV proteins, p-eIF2α, T-eIF2α. (**B**) Quantification of (A). p-eIF2α signal normalized to T-eIF2α and plotted as a ratio. (**C**) EPC cells (1.5 × 10^5^) were treated with GSK2656157 (5 µM) at 1 h prior to infection with rWT VHSV for 12, 24, or 36 h. At indicated time points, cells were pulsed with puromycin (10 µg/mL) for 20 min prior to lysate collection. Cells lysates were separated by SDS-PAGE and immunoblotted with antibodies against puromycin, VHSV proteins, p-eIF2α, and T-eIF2α. (**D**) Quantification of (**C**). p-eIF2α signal and puromycin signal normalized to T-eIF2α and plotted at ratio. (**E**) Media collected at indicated time points post infection were used to determine viral titers. (**F**) RT-qPCR analysis of mRNA levels of VHSV P. Data were normalized to spiked internal control and graphed as fold change normalized to no infection. (**G**) RT-qPCR analysis of IFNβ mRNA levels. Data were normalized to spiked internal control and presented as fold change in expression compared to non-infected. (**H**) Media collected at indicated times post infection were used to measure units of IFN. Error bars reflect SEM *, *p* < 0.05; **, *p* < 0.01; ***, *p* < 0.001.

PKR and PERK are serine/threonine kinases activated in response to viral infection to phosphorylate eIF2α and inhibit translation. The role of PKR or PERK in EPC cells infected with VHSV IVb was determined by treatment without or with inhibitor as indicated and cell lysates were analyzed for phosphorylation of eIF2α [21,22,23]. VHSV-induced phosphorylation of eIF2α was inhibited 1.5-fold with PERK inhibitor treatment compared to untreated VHSV-infected cells (Figure 6A,B). Neither PKR inhibitor altered VHSV-induced phosphorylation of eIF2α, suggesting that phosphorylation of eIF2α during VHSV IVb infection was mediated by PERK and not PKR. Interestingly, we also observed a significant decrease in viral protein expression when PERK was inhibited, suggesting that PERK-induced phosphorylation of eIF2α is required for viral protein synthesis (Figure 6A). Infection of cells with UV-irradiated virus did not induce phosphorylation of e-IF2α, indicating that VHSV IVb viral replication intermediates induce p-eIF2α.

To determine if inhibiting PERK-induced phosphorylation of eIF2α reverses virus-induced repression of host cell protein translation, EPC infected cells treated without or with PERK inhibitor were pulsed with puromycin before harvesting lysates (Figure 6C,D. Phosphorylation of eIF2α, as well as viral protein synthesis, was severely repressed in the PERK inhibited cells compared to the control untreated but infected cells. Despite the reduced p-eIF2α and viral protein synthesis in the PERK inhibitor treated cells, we observed no significant reversal of virus-induced host protein synthesis inhibition, as determined by puromycin incorporation. Treatment with the PERK inhibitor resulted in 1 to 2-log decrease in viral yield, as well as a significant decrease in viral mRNA at the early stages of infection (12 and 24 hpi) (Figure 6E,F). By 36 hpi, there was no significant difference in levels of viral mRNA, despite a significant decrease in viral protein expression (Figure 6C,E,F). Treatment with the PERK inhibitor did not significantly impact interferon production or IFN mRNA levels in infected cells (Figure 6G,H). Our results suggest that VHSV-induced repression of host translation and inhibition of the host immune response is independent of activation of PERK and p-eIF2α and therefore must occur through an alternate regulatory pathway.

## 4. Discussion

The innate immune system acts as the first line of defense against invasion by viral pathogens. In order to survive and propagate, most viruses have developed mechanisms to evade the host immune response. Common strategies used by viruses to evade the host immune response include inhibition of host transcription and translation. This serves to not only inhibit expression of immune response genes, specifically type I IFNs, but also allows the virus to utilize the cell’s translational machinery to synthesize viral gene products. Because of this dependence on the host translational machinery, the innate shutdown of host cell translation serves as a major host defense mechanism against viral infections. Previous studies have identified VHSV IVb M as an anti-host protein that inhibits host antiviral responses by blocking host transcription [24]. However, to date, the impact of VHSV on host translation remains unclear. Our results, in EPC cells, show that overexpression of VHSV IVb NV augments IFN signaling and that infection with VHSV lacking IVb NV resulted in inhibition of the innate immune responses. Despite a small increase in viral RNA with VHSV IVb NV mutant virus infection, we observed a significant decrease in viral protein expression and viral yield, suggesting that IVb NV is required for efficient viral protein synthesis. By measuring puromycin incorporation into nascent proteins, we observed lower levels of newly synthesized proteins in IVb NV deficient VHSV infected cells compared to rWT VHSV infected cells. Further, rWT VHSV infection activated PERK-mediated p-eIF2α, but infection with VHSV IVb NV mutant viruses resulted in decreased p-eIF2α. Finally, we demonstrate that inhibition of PERK-mediated p-eIF2α resulted in decreased viral protein expression but did not rescue host translation or significantly impact IFN signaling. We propose that during VHSV infection, IVb NV is important for PERK-mediated p-eIF2α to promote viral protein synthesis. We also propose that IVb NV regulates the viral shut down of host translation and IFN signaling through a separate pathway.

To identify how VHSV IVb NV manipulates the type I IFN response, we investigated the impact of both VHSV-ΔATG and VHSV NV deletion (VHSV-ΔNV) mutant viruses on IFN and ISG responses. In contrast to other studies demonstrating an inhibition of IFN signaling by VHSV Ia and IVa NV proteins, we demonstrated an augmentation of type I IFN signaling genes by the IVb NV protein, and showed a decrease in IFN mRNA and secretion with deletion of IVb NV or IVb NV-ATG during viral infection (Figure 1D–H). This suggests that the impact of the NV protein on the host immune response may be different between the different VHSV genotypes [25,26,27,28,29]. In this regard, previous studies have shown that VHSV NV evolves faster than either N or G that are used to categorize VHSV genotypes, strains and substrains [6]. At least one recent study showed that even a few NV amino acid changes altered its function significantly [36]. As such, NV variability exceeds that of other VHSV genes, and such changes are likely to impact NV function. This may occur both within the specific genotype or, as suggested here, between genotypes. The MI03GL strain of VHSV IVb has a number of amino acid differences as compared to all other strains or genotypes, and other amino acid changes in common with some, but not all other strains, substrains or genotypes The significance of these differences in NV and its impact on host response remain to be explored. We further showed that the decrease in IFN signaling with IVb NV deficient viral infection was the result of inhibition of the IFN response by IVb NV deficient VHSV (Figure 2B). We propose that VHSV IVb may use NV to help temper the inhibition of IFN signaling, as well as the host shutoff by other viral proteins such as M, in order to help prolong the survival of the cell and ultimately allow for extended viral replication. Previous studies on the impact of VHSV on apoptosis has demonstrated a role of IVb NV in the delay of apoptosis during viral infection and also showed that preventing virus-induced cytopathicity with the addition of a pan caspase inhibition (Z-VAD-FMK) resulted in increased viral yield [31,37].

IVb NV has previously been implicated in efficient viral replication. When investigating which replication steps might be impacted by IVb NV, we did not observe a significant decrease in viral mRNA post infection, suggesting that IVb NV is not required for viral entry to the cell or transcription of the viral genome (Figure 3A). Our results showed instead a decrease in viral protein expression in IVb NV deficient virus infection, suggesting the observed impact on viral replication with removal of IVb NV is due to reduced viral protein synthesis (Figure 3B,C). The observed increase in viral mRNA with NV deficient VHSV infection may be a result of the accumulation of viral mRNAs due to the decrease in viral protein synthesis and will be addressed in future studies. Incorporation of puromycin into infected cells demonstrated a significant decrease in newly translated proteins in IVb NV deficient VHSV infected cells compared to rWT VHSV infected cells (Figure 4A,B), further suggesting a deficiency in viral protein synthesis in IVb NV deficient VHSV infected cells.

To further understand the molecular mechanisms underlying the decrease in translation, we investigated the role of the PKR and PERK pathway, which regulate stress response by phosphorylating eIF2α causing translational arrest [38]. We show that VHSV infection induced p-eIF2α, and as the infection progressed, the amount of p-eIF2α dramatically increased, resulting in host cell translational shutoff as demonstrated by a significant decrease in puromycin incorporation (Figure 5A,B). Interestingly, translation of viral proteins does not seem to be negatively impacted by reduced host protein translation, as viral protein expression continued to increase despite decreased host translational activity (Figure 5A,B). Removal of IVb NV from VHSV resulted in decreased p-eIF2α, implicating a vital role of IVb NV during VHSV infection in the p-eIF2α (Figure 5C,D). Using pharmacological inhibitors, we further identified that phosphorylation of eIF2α was mediated via PERK rather than PKR (Figure 6A,B). When cells were treated with a PERK inhibitor and host translational activity was assessed by puromycin incorporation, we observed no rescue in host cell translational activity (Figure 6C,D). Interestingly, we also observed a significant decrease in viral protein expression as well as a significant decrease in viral yield with the PERK inhibitor treatment (Figure 6C,E). However, PERK inhibitor treatment appeared to have no significant impact on innate immune signaling despite a significant decrease in viral proteins and replication (Figure 6G,H). Virus infections can induce an ER stress response, which results in a switch of the translation initiation from cap-dependent to internal ribosome-entry sites (IRES)-dependent [39]. Often times, viruses, particularly RNA viruses, contain an IRES and therefore will benefit from inducing ER stress by enhancing protein synthesis of their own viral mRNAs [40]. Some viruses, even if they do not contain an IRES, can still initiate cap-independent translation through interaction with ribosomal proteins (RPs) [41,42]. For example, another rhabdovirus, Vesicular Stomatitis virus (VSV), requires RPL40 for VSV mRNA translation, but RPL40 is not required for general cap-dependent protein synthesis [43]. It remains to be explored if VHSV utilizes any of these mechanisms to translate the viral proteins despite shutting off host translation, and if translation factors other than eIF2α are used to mediate these effects. Interestingly, innate immune signaling continues to occur despite a shutoff in host translation during the course of viral infection suggesting that a preferential synthesis of IFN related mRNAs over basal mRNAs by the host cell may be occurring during VHSV IVb infection. Several studies have shown that during viral infection synthesis of type I IFN, other cytokines are preferentially upregulated to allow efficient host response while other cellular protein expression is compromised [44,45]. We observed a significant decrease in viral mRNA with PERK inhibitor treatment at the early stages of infection, but observed no significant difference at later timepoints (36 hpi) despite a significant decrease in viral protein expression (Figure 6C,F). We propose that the inhibition of initial translation of viral proteins early in infection, with PERK inhibitor treatment, caused a delay in viral mRNA accumulation by preventing the activation of the RNA-dependent RNA polymerase (RdRp) that is required for making more viral RNA copies. During VHSV infection, as with other rhabdoviruses, the L protein, which encodes the RdRp, cannot engage the N mRNA directly, but rather depends on the phosphoprotein (P) to facilitate the interaction and if viral protein synthesis is inhibited too early in infection, transcription of viral mRNA can be severely hindered [46]. These observations suggest that the PERK-eIF2α mechanism is activated during VHSV infection but is not solely responsible for the host cell translation shutdown as well as the antagonism of the innate immune response by VHSV. Furthermore, these observations suggest that VHSV requires PERK-mediated p-eIF2α for synthesis of its viral proteins. However, the role of other eIFs in regulating viral vs. host protein translation needs further studies.

Based on our findings, VHSV IVb likely employs a strategy to hijack the PERK-eIF2α pathway to escape widespread inhibition of translation and to efficiently translate its own viral protein. During VHSV infection, the IVb NV protein is required for the PERK-mediated p-eIF2α and efficient viral protein synthesis. The exact mechanism by which IVb NV mediates the PERK-eIF2α pathway during VHSV infection remains unclear and further studies are needed. During VHSV infection, shutdown of host translation and innate immune signaling is independent of the PERK-eIF2α pathway and seems to be regulated by an unknown mechanism. The impact of VSV on host translation during infection has been extensively studied. Studies proposed that VSV alters the eIF4F translation initiation complex to inhibit host protein synthesis and that the abundance of viral mRNA during VSV infection contributes to the host cell shutoff [47,48,49]. VHSV IVb may be impacting host translational shutoff in a similar manner to VSV and further studies to better identify the exact mechanism are required. Furthermore, it will be interesting to examine the role of IVb NV in modulating host gene expression as well as IFN signaling and to determine if IVb NV interacts with other viral products to help mediate translation of host mRNAs and antiviral mRNAs. These approaches will enable dissection of the molecular mechanisms in pathogenicity of VHSV and contribute towards developing attenuated mutant viruses that may be used as immune adjuvants or vaccines to combat the spread of VHSV and related pathogens.

## Figures and Tables

**Table 1:** Primers for cloning.

Primer Name	Sequence (5′→3′)	Restriction Site
VHSV IVbNV-ATG2 se	ACGAATTCTAGACGATCCAGCCGGCACACAGCACAACCAG CTTCTCTCCACTTGTCCTCCACGAGTAGATCGCATAC	EcoR1
VHSV IVbNV as	GGTACCCAGTGAAGGAGACTCAGAGC	Kpn1

**Table 2:** Primers for RT-qPCR.

Primer Name	Sequence (5′→3′)
VHSV IVb N se	ACGGATCCAAAACGCAGATCAG
VHSV IVb N as	AGGGGTGAGTATACAGTGGAGT
VHSV IVb P qRT as	TGTTGTCGGTCTTCTTCCCG
VHSV IVb P qRT se	TGAGGCGTATCAAGCTGTCC
VHSV IVb L qRT se	CCAGTGACCCTCCGGATCTA
VHSV IVb L qRT as	CGATTCCACCCATCAACCCA
VHSV IVb M qRT as	ACTGGGCTTGAGTCCAGGTA
VHSV IVb M qRT se	TCAACCCCCTGGTTCACCTA
VHSV IVb G qRT as	AGCTTTGCTTCCAGGATGGT
VHSV IVb G qRT se	TCCGTTATCAGTCACCAGCG
VHSV IVb NV qRT se	CAGCACAACCAGCTTCTCTCC
VHSV IVb NV qRT as	GTTGAGGTAGTTGCTTGGGTCA
EPC IFN se	TGGGTGGAAAATATCCTGAG
EPC IFN as	CTCCTTATGTGATGGCTGGT
Fish β-actin se	AGACATCAGGGTGTCATGGTTGGT
Fish β-actin as	GGGGTGCTCCTCTGGGGCAA
GFP se	ATGGTGAGCAAGGGCGAGGA
GFP as	TAGCGGCTGAAGCACTGCACGCC
